# Sphingosine-kinase-1 expression is associated with improved overall survival in high-grade serous ovarian cancer

**DOI:** 10.1007/s00432-021-03558-x

**Published:** 2021-03-03

**Authors:** L. C. Hanker, A. El-Balat, Z. Drosos, S. Kommoss, T. Karn, U. Holtrich, G. Gitas, M. Graeser-Mayer, M. Anglesio, D. Huntsman, A. Rody, H. Gevensleben, F. Hoellen

**Affiliations:** 1 Department of Gynecology and Obstetrics, University Hospital Luebeck, Luebeck, Germany; 2 Department of Obstetrics and Gynecology, Goethe-University Frankfurt, Frankfurt, Germany; 3 Department of Woman’s Health, Tuebingen University Hospital, Tuebingen, Germany; 4 Evangelical Hospital Bethesda, Lower Rhine Breast Center, Moenchengladbach, Germany; 5 Department of Molecular Oncology, BCCA Cancer Research Centre, Vancouver, Canada; 6 Institute of Pathology, University Hospital Bonn, Bonn, Germany

**Keywords:** SPHK1, Ovarian cancer, Prognosis

## Abstract

**Purpose:**

Sphingosine-kinase-1 (SPHK1) is a key enzyme of sphingolipid metabolism which is involved in ovarian cancer pathogenesis, progression and mechanisms of drug resistance. It is overexpressed in a variety of cancer subtypes. We investigated SPHK1 expression as a prognostic factor in epithelial ovarian cancer patients.

**Methods:**

Expression analysis of SPHK1 was performed on formalin-fixed paraffin-embedded tissue from 1005 ovarian cancer patients with different histological subtypes using immunohistochemistry. Staining intensity of positive tumor cells was assessed semi-quantitatively, and results were correlated with clinicopathological characteristics and survival.

**Results:**

In our ovarian cancer collective, high levels of SPHK1 expression correlated significantly with complete surgical tumor resection (*p* = 0.002) and lower FIGO stage (*p* = 0.04). Progression-free and overall survival were further significantly longer in patients with high-grade serous ovarian cancer and overexpression of SPHK1 (*p* = 0.002 and *p* = 0.006, respectively).

**Conclusion:**

Our data identify high levels of SPHK1 expression as a potential favorable prognostic marker in ovarian cancer patients.

**Supplementary Information:**

The online version of this article (10.1007/s00432-021-03558-x) contains supplementary material, which is available to authorized users.

## Background

Epithelial ovarian cancer (EOC) remains the fifth most common lethal cancer in women. The unfavorable prognosis of this disease is associated with advanced stage at first diagnosis (Hennessy et al. 2009). Apart from clinical stage, histological subtypes differ regarding prognostic values. High-grade serous carcinoma represents the largest histological subgroup (68%), followed by clear cell (12%), endometrioid (11%), mucinous (3%) and low-grade serous carcinoma (3%) as well as other rare types 3% (Kobel et al. 2010). The identification of prognostic and predictive biomarkers and especially of molecular targets for individual targeted cancer therapies offers new therapeutic strategies for ovarian cancer patients, particularly with regard to chemotherapy resistance. (Kobel et al. 2013). Despite high response rates to chemotherapy in ovarian cancer, drug resistance occurs frequently. Consequently, approximately 80% of patients with International Federation of Gynecology and Obstetrics (FIGO) stage II–IV EOC will progress during or after first-line adjuvant chemotherapy (du Bois et al. 2009). The need for individualized treatment strategies based on biologically distinct subgroups is evident, especially in platinum-resistant recurrent disease.

A variety of different signaling pathways and their respective metabolites have been associated with mechanisms of tumor pathogenesis and progression, and the expression of these metabolites is characteristic of individual cancer subtypes. Sphingolipid metabolism is one of these pathogenetic pathways involved in both inflammatory and oncological disease. Sphingolipids are an integral part of the cellular membrane and exert a regulatory role in cellular functions, e.g., apoptosis and cell proliferation. We have previously demonstrated the correlation of acid ceramidase expression, another key enzyme of sphingolipid metabolism, with a favorable prognosis in OC (El-Balat et al. 2020; Hanker et al. 2013). Ceramide and sphingosine mediate and trigger apoptosis and cell growth arrest, whereas sphingosine 1-phosphate (S1P) enhances proliferation and cell survival. The balance between the intracellular levels of these sphingolipids is controlled by the key enzymes which produce or degrade these metabolites. Ceramide is converted into sphingosine that, in turn, is phosphorylated by a sphingosine kinase (SPHK; two isoforms SPHK1 and SPHK2) to form S1P. During this process, phosphorylation of sphingosine is a rate-limiting step in the sphingolipid metabolism. The activity of SPHK is crucial for maintaining the balance between proapoptotic and prosurvival signaling lipids (Guillermet-Guibert et al. 2009). Intracellular S1P is generated by SPHK and then secreted into the extracellular milieu where it activates cell surface S1P receptors leading to the activation of downstream signals (Dai et al. 2017). S1P contributes to cancer progression by regulating tumor proliferation, invasion and angiogenesis. In vitro studies have shown that neutralizing S1P with anti-S1P monoclonal antibodies inhibits neovascularization in multiple tumor cell lines which represents an impact that is less likely to be bypassed by production of alternative proangiogenic factors targeting tumor vessels (Visentin et al. 2006). S1P is aberrantly expressed in ovarian cancer patients, and serum S1P levels have been shown to be elevated in ovarian cancer patients and decreased after tumor surgery. Moreover, S1P is involved in the regulation of key cellular processes that contribute to ovarian cancer initiation and progression. Inhibition of the S1P signaling pathway has been proposed to inhibit ovarian cancer cell growth and induce apoptosis. Consequently, S1P has been suggested as a potential molecular target for ovarian cancer therapy (Dai et al. 2014; Dai et al. 2017).

Recently, SPHK1 has attracted increasing attention because of its important functions in many processes of cancer cells (Liu et al. 2019; Long et al. 2016; Maceyka et al. 2012). SPHK1 is one of the central enzymes of sphingolipid metabolism, and it is a crucial regulator of sphingolipid equilibrium. Its overexpression is presumed to be correlated with carcinogenesis and inflammation (Gupta et al. 2019). On the one hand, SPHK1 favors survival through increasing intracellular levels of the prosurvival sphingolipid metabolite S1P. On the other hand, SPHK1 reduces levels of both ceramide and sphingosine, the proapoptotic sphingolipids (Guillermet-Guibert et al. 2009). As a regulator of cell survival and cell death, the *SPHK1* gene was found to be of oncogenic nature. SPHK1 mRNA is overexpressed in various types of solid tumors, protecting cells from apoptosis and showing decreased activity during anticancer drug therapies (Cuvillier 2007). There is a body of evidence for SPHK1 overexpression in a variety of solid cancers, e.g., prostate cancer, colorectal cancer and non-small-cell lung cancer (Kawamori et al. 2006). Therefore, SPHK1 might represent a new target of interest in terms of pharmacological inhibition in OC. By increasing the ceramide and/or sphingosine content of tumor cells and targeted blocking of sphingosine 1-phosphate tumor cells could possibly be degraded (Cuvillier 2007).

To conclude, the concept that SPHK1 inhibition in combination with chemotherapeutic therapies might sensitize resistant cancer cells to currently inefficient regimens has emerged (Guillermet-Guibert et al. 2009). We, therefore, investigated SPHK1 expression in cancer tissues of 1005 ovarian cancer patients with different histologic subtypes.

## Methods

Retrospective immunohistochemical analysis of SPHK1 expression was conducted on tissues of 1005 ovarian cancer patients who underwent surgery. Immunohistochemical results were associated with patients’ clinicopathological characteristics, prognostic markers and survival.

### Patients and treatment

The study included formalin-fixed paraffin-embedded tissue from 1005 patients with primary EOC. Three previously published independent patient cohorts were analyzed together (Kalloger et al. 2011; Kobel et al. 2008).

The first cohort (termed OOU, i.e., “Ovarian outcome unit”) originally consisted of 540 patients treated between 1984 and 2003 at one of the 20 hospitals which were part of the Cheryl Brown Ovarian Outcome Unit network in British Columbia (Canada). Only patients with ovarian cancer and primary debulking surgery (PDS) resulting in macroscopic complete resection were included. Interval debulking surgery and neoadjuvant chemotherapy were exclusion criteria.

The second patient cohort, termed OOUE (“Ovarian outcome unit extreme risk”), encompassed 250 patients who had been treated at five centers in Canada between 2006 and 2009. This cohort consisted of patients with high-risk ovarian cancers. Only patients with macroscopical residual tumor after PDS were included.

The third cohort (termed VOA) was collected between 2001 and 2008. It was composed of samples of the Gynaecologic Tissue Bank of the Vancouver General Hospital from 316 patients with ovarian cancer who underwent surgical cytoreductive therapy. In contrast to the OOU and OOUE cohorts, patients were included irrespective of surgical results, meaning patients with suboptimal surgery (any residual tumor) and optimal surgery (no residual tumor) were included.

Formalin-fixed paraffin-embedded (FFPE) tissue samples were reviewed by pathologists and only included in our study if histologic results were congruent, if clinicopathological data were sufficient including appropriate follow-up, if staining was adequate and if histological validation showed sufficient tissue with adequate numbers of tumor cells. As a result, a total of 1005 of the 1105 patients could be retrospectively analyzed.

Clinical follow-up data for all patient cohorts were provided by the Cheryl Brown Ovarian Cancer Outcomes Unit as an ovarian cancer database of the British Columbia Cancer Agency (BCCA). Study approval was acquired from the Research Ethics Board of the University of British Columbia. All Local Research Ethics Committees approved studies of human tissue and the samples were processed anonymously. Clinical outcome was assessed from the date of first diagnosis to the date of relapse or death.

### Tissue samples and immunohistochemistry

**Fig. 1:**
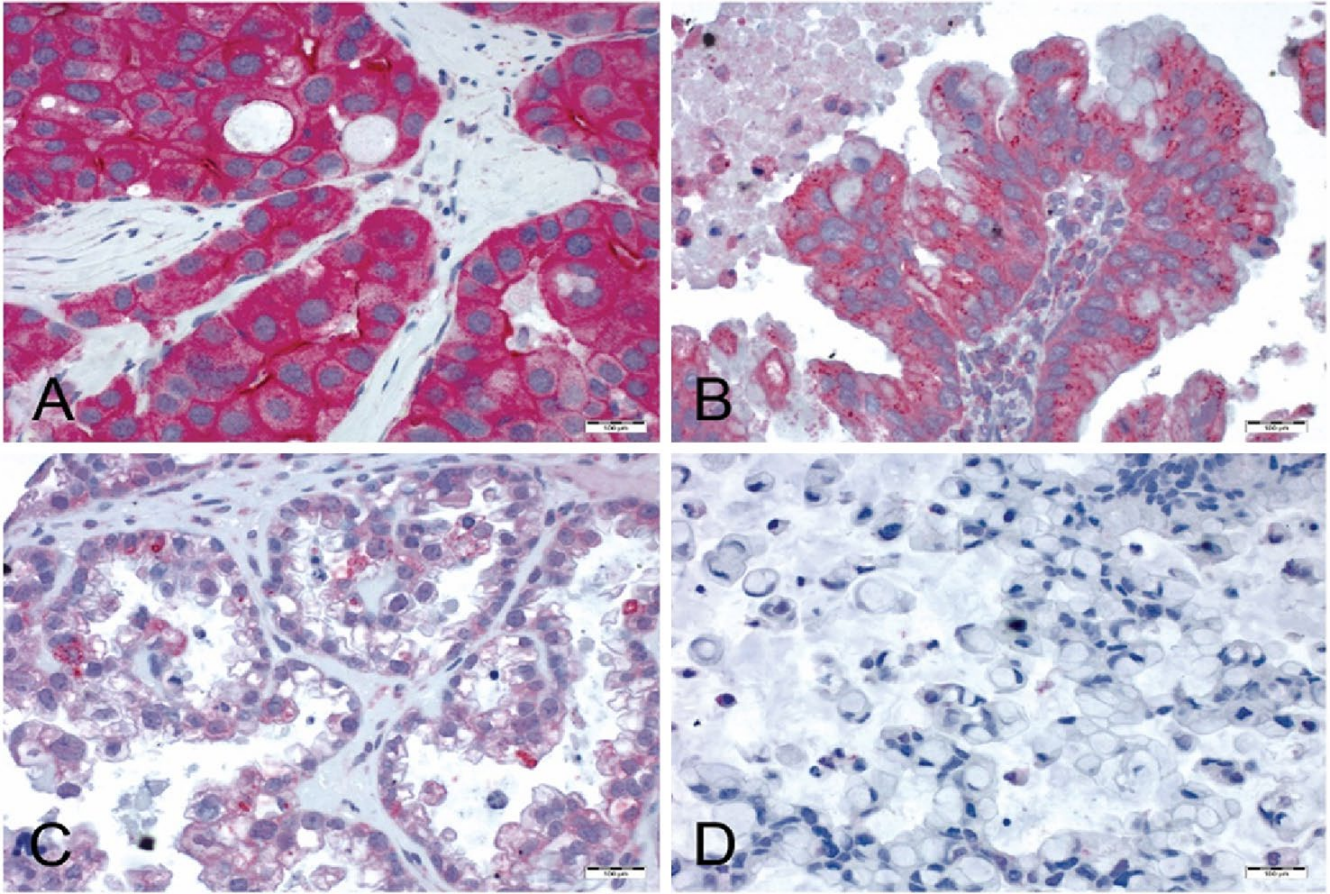
Immunohistochemical staining of SPHK1. Semi-quantitative scoring of cytoplasmic SPHK1 expression in ovarian cancer: A = strong SPHK1 expression, B = medium SPHK1 expression, C = weak SPHK1 expression, D = negative

Tissue samples were processed as previously described (Hanker et al. 2013; Ruckhaberle et al. 2008). All samples underwent contemporary gynecopathological review including predictions of an IHC-based Calculator of Ovarian Carcinoma Subtype (COSP), and a tissue microarray (TMA) was available through earlier studies (Kommoss et al. 2013). Paraffin sections of TMAs were dewaxed in xylene and rehydrated with graduated ethanol treatment. For antigen retrieval, sections were incubated for 20 min in a microwave oven (800 W) using citrate buffer (10 mM; pH 6.0). The SPHK1 antibody (polyclonal peptide affinity-purified SPHK1 antibody, IMGENEX, catalog no. IMG-72025) was used at a dilution of 1:400. Incubation with 200 µl antibody-solution per section for 12 h at 4°Celsius was performed. For negative controls, the primary antibody was omitted. For secondary antibody incubation, the Dako REAL Detection System Alkaline Phosphatase/RED (Dako, Denmark, REF K5005, Lot 20,023,341) was applied following the instructions of the vendor. Sections were subsequently counterstained with hematoxylin (Gill No. 3, Lot 060M4356; Sigma-Aldrich St. Louis, Missouri, United States). Cytoplasmic SPHK1 expression was scored semi-quantitatively based on the staining intensity (SI). SI was assigned as 0, negative; 1, weak; 2, medium; or 3, strong (Fig. 1). All assessments were performed blinded with respect to clinical patient data. Only sections including at least 200 tumor cells were scored.

### Statistical analysis

For statistical analysis, a cut-off value was defined according to the staining intensity (SI), i.e., scores of 0–2 (negative and medium) were collectively defined as a low score; whereas, a score of 3 was defined as a high SPHK1 expression score. The Chi-square and Fisher’s exact tests were used to assess the associations between SPHK1 expression of tumors and clinicopathological parameters. For those patients with available follow-up data, Kaplan–Meier curves were constructed, and the log rank test was used to determine the univariate significance of the variables. Cox regression analysis was performed to determine hazard ratios. All reported P values are two sided and *p* values ≤ 0.05 were considered statistically significant. All analyses were performed using the SPSS software package, version 24.0 (IBM SPSS, Armonk, NY, USA).

## Results

### Patients’ characteristics

Patient characteristics are listed in Table 1. A total of 1005 patients were included in the overall cohort of our study originating from the above-mentioned three single cohorts from British Columbia, as previously described (Kalloger et al. 2011; Kobel et al. 2008). Only patients with epithelial ovarian cancer and available information were included in our study, leading to the exclusion of 101 patients from the original cohorts because of non-epithelial OC histology or missing IHC data. Above all these cohorts differed with respect to the percentage of optimally tumor debulked patients. The OOU cohort added 508 ovarian cancers to the overall cohort, all optimally debulked (i.e., no postoperative residual tumor). In contrast the OOUE cohort added 241 patients which were sub-optimally debulked (i.e., any residual tumor) and the VOA cohort lastly added further 256 cases irrespective of the result of the PDS (i.e., optimal and suboptimal debulking surgery) to the overall cohort. Further details of the composition of each cohort are listed in table S1. All patients included underwent primary debulking surgery and were chemotherapy naïve. In the majority of patients (n = 598; 59.5%), optimal debulking according to the definition of the National Comprehensive Cancer Network guidelines (no postoperative residual tumor) could be achieved. Nevertheless, there is a strong bias because of the sampling strategy of the three different cohorts.

**Table 1:** Patients’ characteristics

Patients’ characteristics	*n* (%)
Overall	1005 (100)
Debulking surgery	
Optimal^$^	598 (59.5)
Suboptimal^§^	407 (40.5)
Age (years)	
Median	58.00
Average	58.92
Range	19–91
FIGO stage	*N* = 1001 (100)
I	261 (26.1)
II	249 (24.9)
III	444 (44.4)
IV	47 (4.7)
Histological subtype	
High-grade serous OC	613 (61.0)
Clear cell OC	165 (16.4)
Endometrioid OC	155 (15.4)
Mucinous OC	46 (4.6)
Low-grade serous OC	20 (2.0)
Transitional cell OC *	6 (0.6)

^*^according to former WHO classification 3^rd^ edit. (until 2014)

^$^no residual tumor

^§^any residual tumor

### SPHK1 expression according to different clinicopathological characteristics

Immunohistochemical analysis revealed high expression levels of SPHK1 in 248 (24.7%) tumor samples and low expression levels in 757 samples (75.3%). In the cohort of 1005 patients, no significant difference in SPHK1 expression was found based on age (< 50 vs ≥ 50 years) or serous histology (high-grade serous vs. other). Low levels of SPHK1 expression were mainly present in the histological subtype of clear cell ovarian cancer with 88.5% of all clear cell OC revealing low SPHK1 expression (Table 2). High SPHK1 levels were associated with complete tumor resection (*p* = 0.002 and lower FIGO stage (*p* = 0.04).

**Table 2:** Tumor characteristics

Tumor characteristics	Low SPHK1 expression, *n* (%)	High SPHK1 expression, *n* (%)	*p*	Overall (*n* = 1005)
Histology			< 0.001	
Clear cell OC	146 (88.5)	19 (11.5)		165
Endometrioid OC	106 (68.4)	49 (31.6)		155
High-grade serous OC	462 (75.4)	151 (24.6)		613
Mucinous OC	24 (52.2)	22 (47.8)		46
Low-grade serous OC	14 (70.0)	6 (30.0)		20
Other*	5 (83.3)	1 (16.7)		6
			0.94^**^	
High-grade serous OC	463 (75.4)	151 (24.6)		614
Other	294 (75.2)	97 (24.8)		391
Age (years)			0.671^**^	
< 50	184 (74.2)	64 (25.8)		248
> 50	573 (75.7)	184 (24.3)		757
Debulking surgery			0.002^**^	
Optimal^$^	430 (71.9)	168 (28.1)		598
Suboptimal^§^	327 (80.3)	80 (19.7)		407
FIGO stage			0.04^**^	n = 1001
I–II	370 (72.5)	140 (27.5)		510
III–IV	384 (78.2)	107 (21.8)		491

^*^Former Transitional cell OC according to WHO classification

^**^Fischer exact test

^$^No residual tumor

^§^Any residual tumor

### Association of SPHK1 expression with survival

**Fig. 2:**
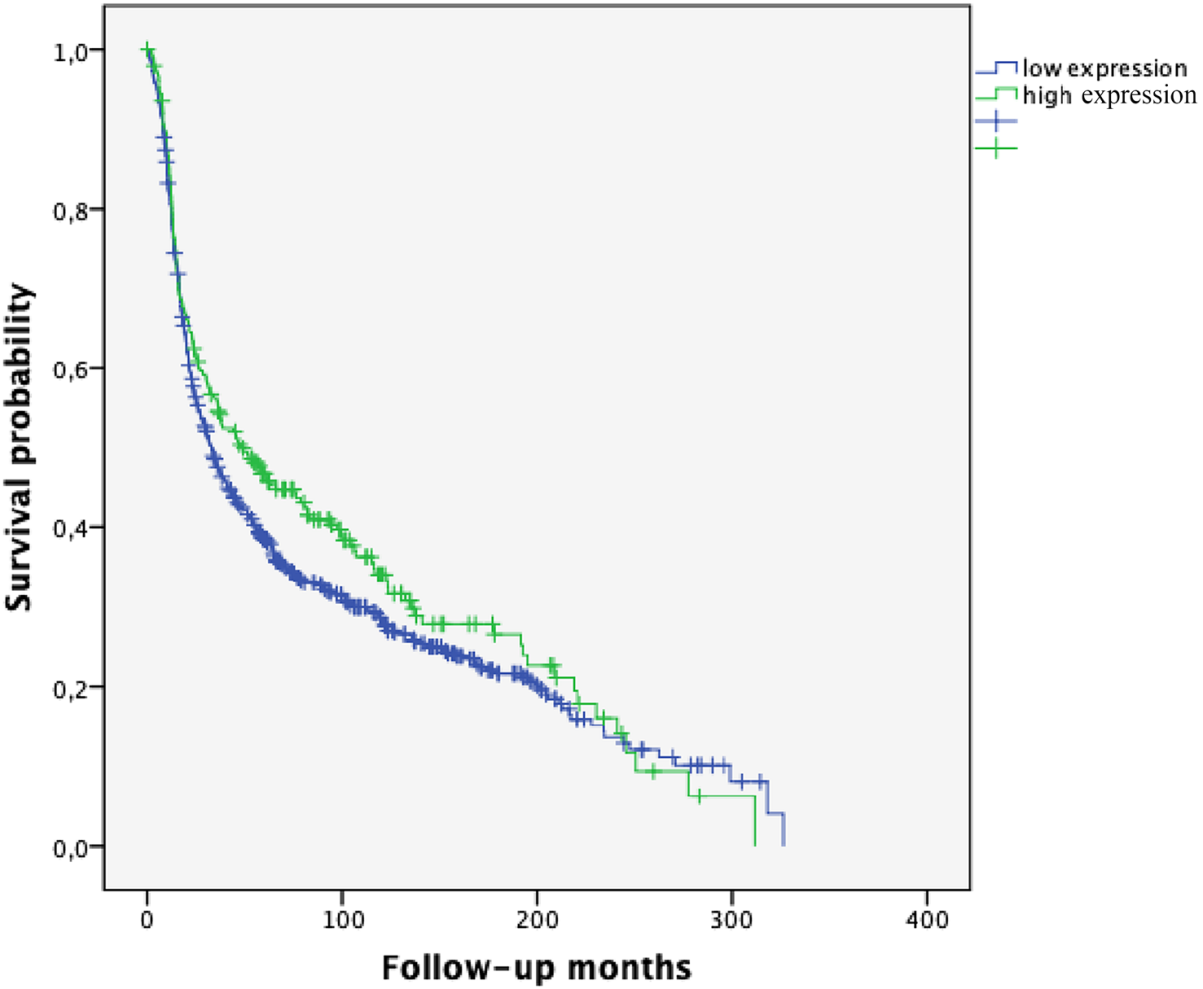
Progression-free survival (PFS) in ovarian cancer patients according to SPHK1 expression (*p* = 0.107; high SPHK1 expression *n* = 248; low SPHK1 expression *n* = 756)

**Fig. 3:**
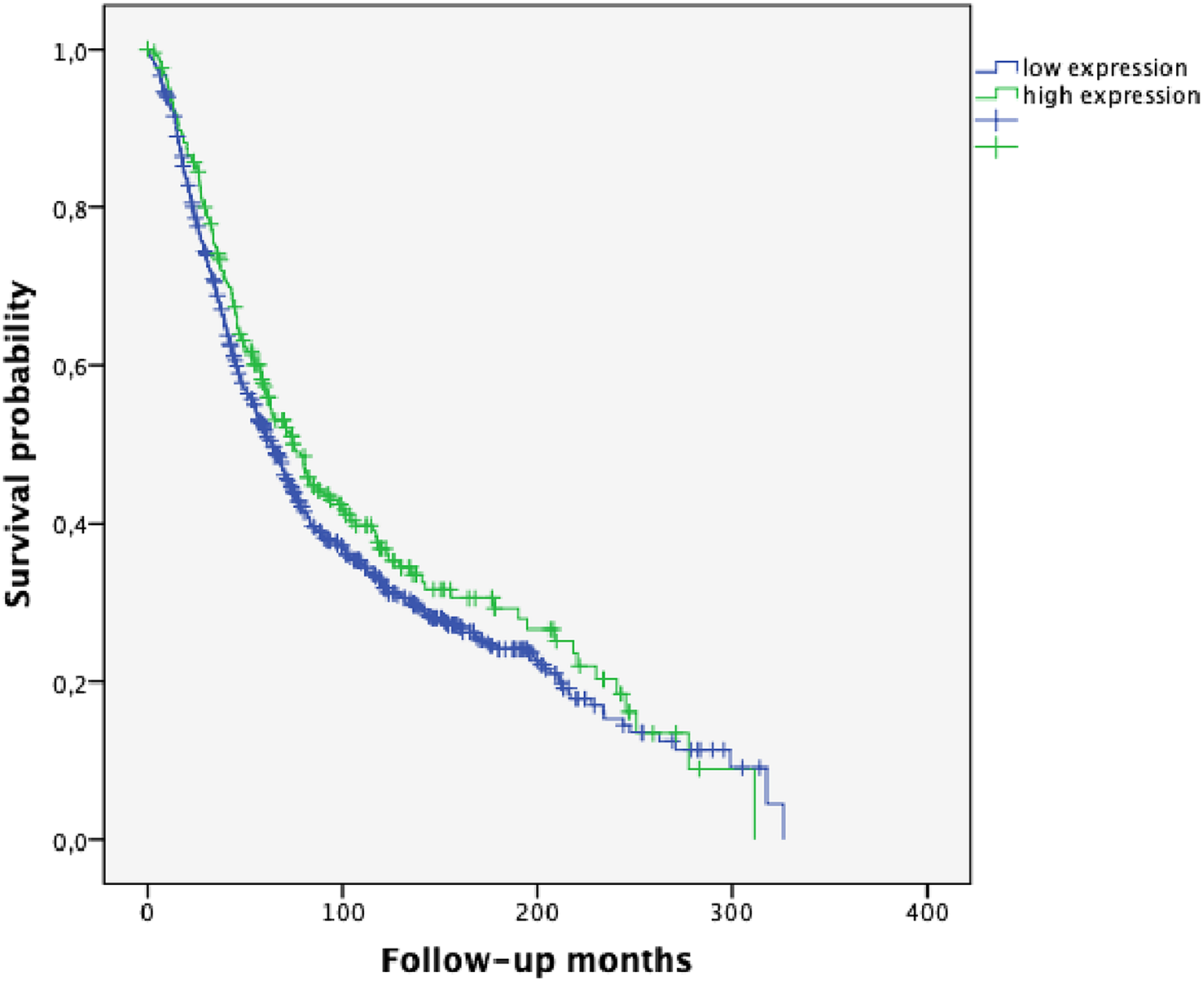
Overall survival (OS) in ovarian cancer patients according to SPHK1 expression (*p* = 0.124; high SPHK1 expression *n* = 248; low SPHK1 expression *n* = 756)

For the entire cohort with available follow-up (*n* = 1004), the Kaplan–Meier estimate for median progression-free survival (PFS) was 35.38 months [95% confidence interval (CI) 29.80–40.95] and for overall survival (OS) 65.65 months (95% CI 59.37–71.94). Kaplan–Meier analysis further showed longer PFS (48.63 months [95% confidence interval (CI) 30.58–66.40] vs. 32.84 months [95% CI 27.52–38.17], *p* = 0.107, Fig. 2) and longer OS (75.35 months [95% CI 62.67–88.03] vs. 64.27 months [95% CI 57.71–70.683], *p* = 0.124, Fig. 3) in OC with high SPHK1 expression.

Univariate Cox-Regression Analysis revealed a highly significant prognostic impact of SPHK1 expression in the subgroup of high-grade serous ovarian carcinoma (HGSOC). High expression was associated with longer PFS and OS (PFS: Hazard ratio (HR) = 1.37 [95% CI 1.12–1.69], *p* = 0.003; OS: HR = 1.34 [95% CI 1.09–1.66], *p* = 0.006).

### Multivariate Cox regression analysis

Multivariate Cox regression analysis for PFS and OS included SPHK1 expression, age (> 50 vs. ≤ 50 years), FIGO stage (I and II vs. III and IV), histology (HGSOC vs. others) and residual tumor (0 vs. > 0 cm). FIGO stage, histology and residual tumor exhibited a statistically significant correlation with poor PFS (all *p* < 0.001) and OS (all *p* < 0.001). In the multivariate Cox regression analysis of the entire cohort, SPHK1 expression did not retain its significant impact on PFS and OS (*p* = 0.78 and *p* = 0.68, Tables 3 and 4, respectively).

**Table 3:** Multivariate cox regression analysis for progression-free survival (PFS)

PFS	*n* = 1001	HR	95% CI		*p*
SPHK1 expression (low vs. high)	754 vs. 247	1.025	0.861	1.221	0.778
Age (< 50 vs. ≥ 50)	247 vs. 754	0.879	0.729	1.060	0.177
FIGO stage (FIGO III–IV vs. I–II)	491 vs. 510	2.025	1.587	2.584	< 0.001
Histology (others vs. serous high-grade OC)	390 vs. 611	0.672	0.547	0.825	< 0.001
Residual tumor (optimal vs. suboptimal)	596 vs. 405	0.415	0.329	0.522	< 0.001

**Table 4:** Multivariate cox regression analysis for overall survival (OS)

OS	*n* = 1001	HR	95% CI		*p*
SPHK1 expression (low vs. high)	754 vs. 247	1.039	0.868	1.244	0.676
Age (< 50 vs. ≥ 50)	247 vs. 754	0.824	0.678	1.002	0.052
FIGO stage (FIGO III–IV vs. I–II)	491 vs. 510	2.086	1.628	2.672	< 0.001
Histology (others vs. serous high-grade OC)	390 vs. 611	0.683	0.552	0.845	< 0.001
Residual Tumor (optimal vs. suboptimal)	596 vs. 405	0.532	0.423	0.670	< 0.001

### Association of SPHK1 expression with survival in high-grade serous ovarian carcinoma (HGSOC)

**Fig. 4:**
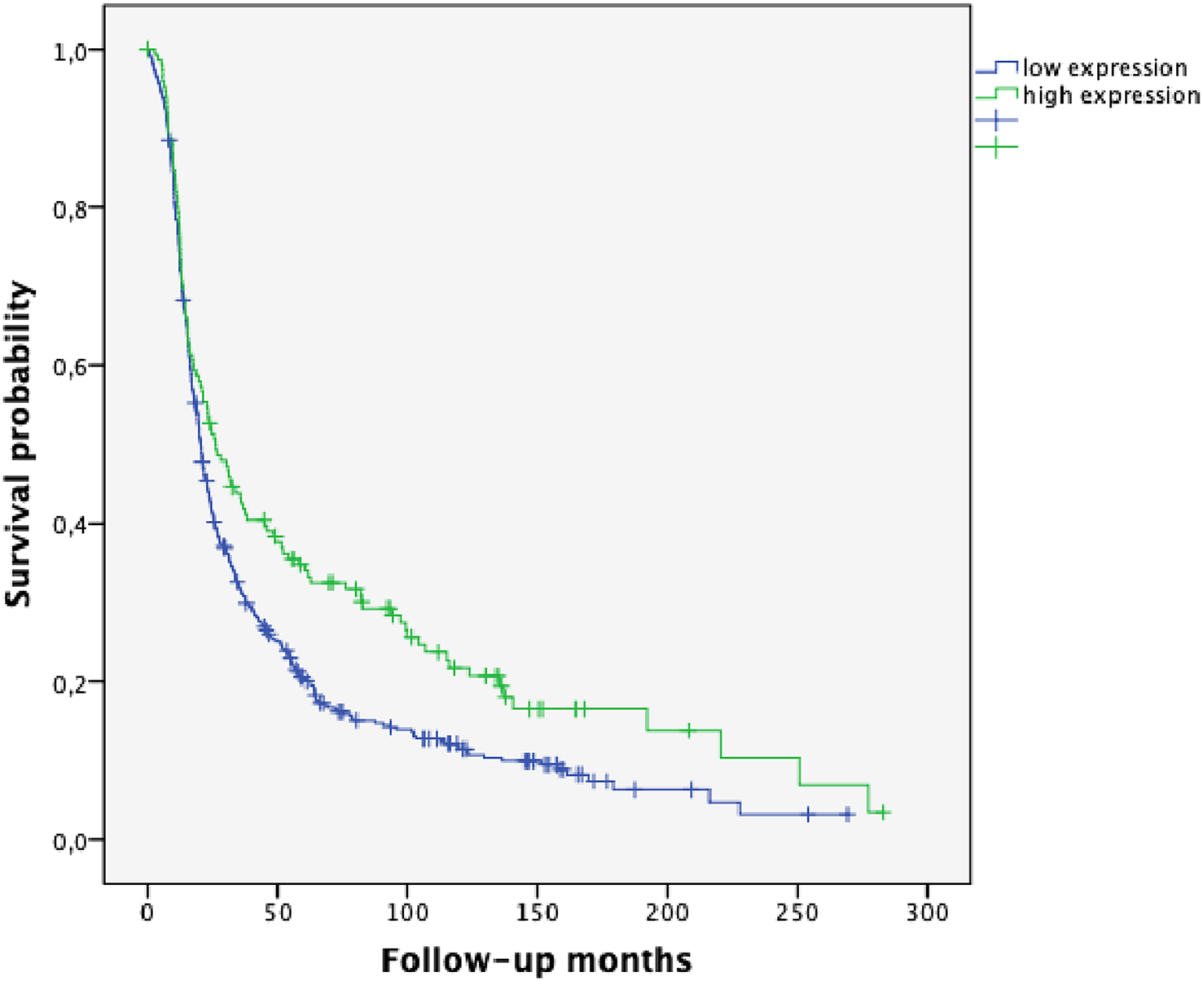
Progression-free survival (PFS) in high-grade serous carcinoma (HGSOC) according to SPHK1 expression (*p* = 0.002; high SPHK1 expression *n* = 151; low SPHK1 expression *n* = 461)

**Fig. 5:**
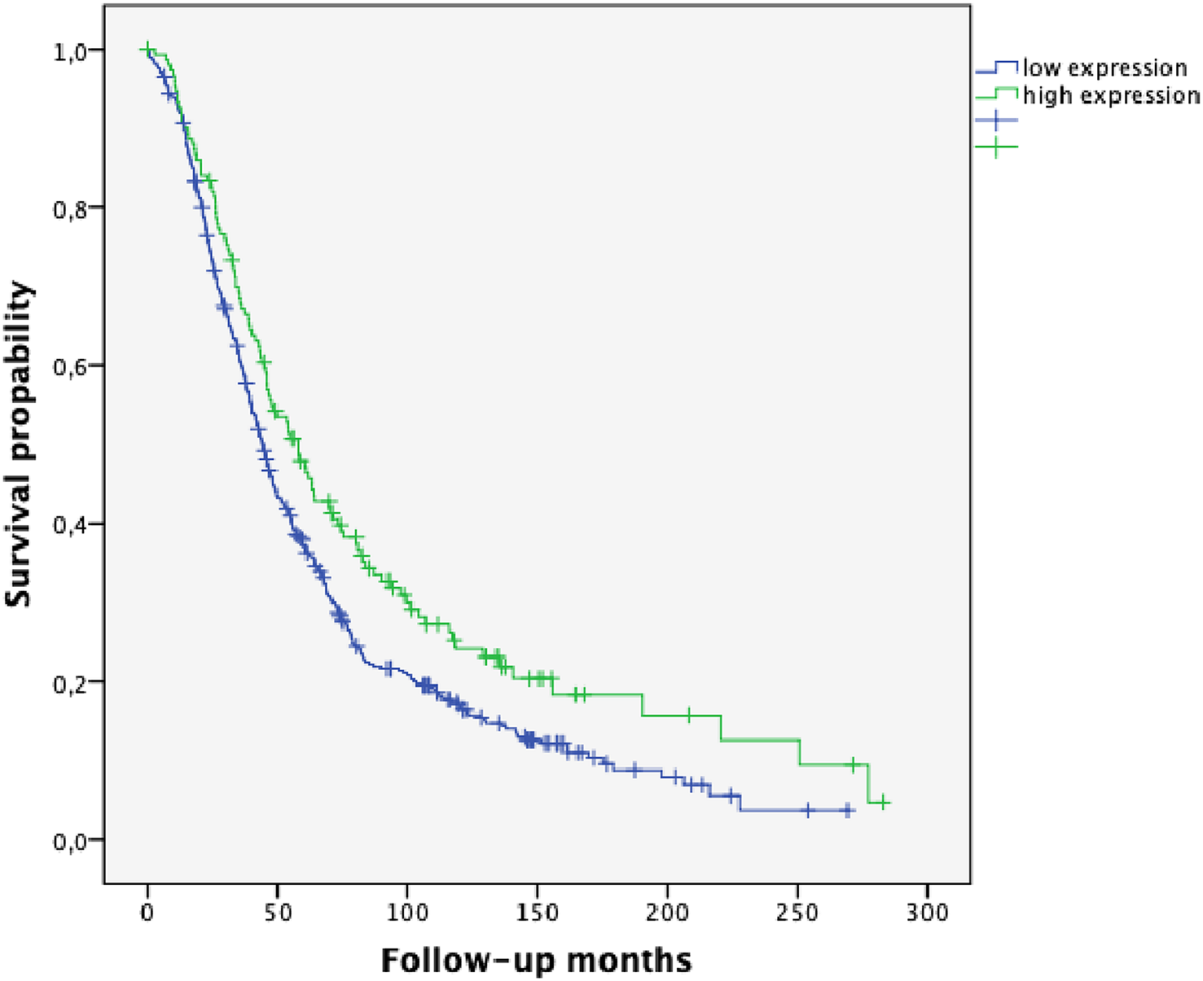
Overall survival (OS) in high-grade serous carcinoma (HGSOC) according to SPHK1 expression (*p* = 0.006; high SPHK1 expression *n* = 151; low SPHK1 expression *n* = 461)

HGSOC represented the most frequent histological subgroup in our study (n = 613, 61.0%). 75.4% of the cases in this subgroup showed low SPHK1 expression, whereas 24.6% exhibited high SPHK1 expression (Table 1). In the Kaplan–Meier analysis, high SPHK1 expression was associated with significantly longer PFS and OS (*p* = 0.002 and *p* = 0.006, Figs. 4, 5, respectively). In HGSOC with high SPHK1 expression, the median PFS was 26.30 months (95% CI 18.28–34.32) vs. 20.38 months in SPHK1 low expression cases (95% CI 18.33–22.44). The median OS was 58.06 months in SPHK1 high expression HGSOC (95% CI 45.91–70.21) vs. 44.12 months in SPHK1 low expression cases (95% CI 40.24–48.00).

### Association of SPHK1 expression with survival in clear cell carcinoma (CCC)

In the subgroup of clear cell carcinoma (*n* = 165, 16.4%), low SPHK1 expression was detected significantly more often (low SPHK1 in 88.5%, high SPHK1 in 11.5%, Table 1) than in all other histological subtypes (*p* < 0.001). Nevertheless, Kaplan–Meier analysis of the subgroup of clear cell ovarian carcinomas (*n* = 165) did not reveal any distinct trend for better PFS or OS in patients with high SPHK1 expression (*p* = 0.754 and *p* = 0.474, respectively).

### Association of SPHK1 expression with survival in endometrioid ovarian carcinoma

In the subgroup analysis of endometrioid ovarian cancer (n = 155, 15.4%), low SPHK1 expression was detected more often than high expression (low SPHK1 in 68.4%, high SPHK1 in 31.6%). There was also no significant correlation with either PFS or OS according to SPHK1 expression levels in the Kaplan–Meier analysis (*p* = 0.421 and *p* = 0.720, respectively).

## Discussion

The SPHK1 receptor signaling pathway has been implicated in a variety of pathological processes of ovarian cancer in vitro (Dai et al. 2017). SPHK1 overexpression, for instance, has been described in different cancer subtypes both in in vivo and in vitro studies, and its role in tumor initiation, progression (e.g., angiogenesis) and metastasis has been shown, e.g., in lung cancer, breast cancer, pancreatic cancer and colorectal cancer (Acharya et al. 2019; Guillermet-Guibert et al. 2009). Furthermore, the aspect of drug resistance via sphingolipid metabolism-associated pathways has been described. Molecular markers like SPHK1 can potentially serve as diagnostic as well as prognostic and predictive markers. To our knowledge, this is the first report on an association of prognosis and SPHK1 in ovarian cancer.

Surgical outcome in ovarian cancer is usually classified according to the amount of postoperative residual tumor. A complete resection is regarded if no macroscopically visible tumor is left. The prognostic value of complete optimal debulking has been reported on several occasions and has been confirmed in a metaanalysis (du Bois et al. 2009). In accordance, both PFS and OS were significantly better in patients with optimal surgery in our patient collective (p < 0.05). Overall, our patient collective showed a comparatively high percentage of early-stage EOC (FIGO I and II) and subsequently relatively long PFS and OS compared to stages at first diagnosis and survival data expected in general populations of ovarian cancer patients (du Bois et al. 2009). This fact might be due to the inclusion criteria and clinical characteristics of the OUU collective, which only included patients with complete surgical tumor resection. Consequently, patients with higher FIGO stages III and IV who were not appropriate for complete resection were not included. Although the VOA and OOUE cohort also included patients with residual tumor burden, the proportion of these patients in our cohort is lower than one would expect. The primary eligibility criterion in the OUU collective was the diagnosis of chemotherapy-naive ovarian carcinoma, with surgical debulking leading to patients free of macroscopic residual disease after primary cytoreductive surgery. As a result of this case definition, there were a relatively large number of non-serous carcinomas compared to what would be expected from a series including all patients with ovarian cancer (Kalloger et al. 2011).

Ovarian carcinoma is composed of five major histologic subtypes which associate with outcome and predict therapeutic response. Thus, reliable identification of histotypes is essential for the success of studies testing novel therapies as well as for biomarker discovery research (Kommoss et al. 2013). Emerging evidence points out that the five major ovarian carcinoma subtypes (high-grade serous, clear cell, endometrioid, mucinous and low-grade serous) are distinct disease entities (Kalloger et al. 2011). An association of optimal tumor surgery with histopathologic subtype has been described in the literature (Mackay et al. 2010). In our study, HGSOC correlated with suboptimal tumor surgery and, thus, worse survival; whereas, high SPHK1 expression was associated with optimal tumor debulking. In the present study, Kaplan–Meier analysis of PFS and OS revealed a strong trend for better prognosis for patients with high SPHK1 expression (*p* = 0.107 and *p* = 0.124, Figs. 2 and 3, respectively). However, these findings were not significant when analyzing the whole group of patients encompassing all major different histological subtypes. By considering the histological subgroups separately, a strong prognostic impact of high SPHK1 expression could be seen in HGSOC. In this subgroup, representing the most frequent histological subtype, we could demonstrate a highly significant longer PFS (*p* = 0.002, Fig. 4) and OS (*p* = 0.006, Fig. 5) in patients with overexpression of SPHK1. However, these findings were not retained in multivariate COX regression analysis. To a certain extent, this observation might be attributed to the strong association of the absence of postoperative residual tumor and overexpression of SPHK1. Therefore, the impact of SPHK1 expression on PFS and OS does not seem to be independent. Nevertheless, it should be further evaluated as possible marker for surgical outcome.

While there was no conclusive correlation of HGSO with SPHK1 expression in our patient collective, we found a distinct correlation of low SPHK1 expression with the histological subtype of clear cell carcinoma. Furthermore, low SPHK1 expression correlated with incomplete resection and thus, in accordance to the literature, with a lower survival rate. Ovarian clear cell carcinoma subtype represents approximately 5–10% of all EOCs (Lalwani et al. 2011). In our patient collective, clear cell carcinoma subtype occurred relatively often with 16.4% (see Table 2). Nevertheless, a distinct trend for a better PFS and OS could not be pointed out for high expression of SPHK1 in the subgroup of clear cell carcinoma.

The subgroup analysis of endometrioid ovarian cancer in our collective did not show a significant difference for OS or PFS with regard to SPHK1 expression. Interestingly, we recently showed that the expression of acid ceramidase (ASAH1), another key player in sphingolipid metabolism and signaling, was associated with significantly improved overall survival in estrogen receptor-negative endometrioid ovarian cancer. Further studies are needed to differentiate between estrogen receptor-negative and -positive subtypes of endometrioid ovarian cancer to elucidate the association with SPHK1 expression and survival. Our findings support the hypothesis that management of ovarian carcinoma will become subtype specific in the future, and that sphingolipid metabolism-associated factors might play a role in terms of subtype-specific individual therapeutic strategies.

Interestingly, Zhu et al. reported on longer PFS and OS in breast cancer patients with low expression of SPHK1 and a significant association of high SPHK1 expression with poor prognostic parameters (presence of lymph node metastasis, number of positive lymph nodes and presence of distant metastasis). In breast cancer, high SPHK1 expression was also associated with human epidermal growth factor receptor 2 (HER2) status but not with tumor histological subtypes, histological grade, tumor size or hormone receptor status (Zhu et al. 2017). Poor oncologic prognosis in association with SPHK1 expression has also been reported in cervical cancer and non-Hodgkin lymphoma. Beyond its prognostic impact, it could also serve as an anticancer therapeutic target (Gao et al. 2015).

In a variety of cancer subtypes, SPHK1 expression has been associated with drug resistance. SPHK1 is overexpressed in triple-negative breast cancer (TNBC) and promotes metastasis. Targeting SPHK1 or its downstream target NFκB with clinically available inhibitors could, therefore, be effective for treating metastasized TNBC (Acharya et al. 2019). In pancreatic cancer, targeting the sphingolipid metabolism for improving tumor chemosensitivity has been proposed as a promising strategy. Inhibitors of SPHK1 are used in ongoing clinical trials to sensitize epithelial ovarian and prostate cancer cells to various chemotherapeutic drugs, e.g., gemcitabine (Guillermet-Guibert et al. 2009). In the non-small-cell lung cancer cell line A549, inhibition of SPHK1 by its specific inhibitor SKI-II increases the sensitivity of the cells to paclitaxel. Thus, SPHK1 expression might play a crucial role in drug resistance in these cancer cells (Wu et al. 2019). Furthermore, insulin-like growth factor-1 (IGF-1)-induced epithelial–mesenchymal transition (EMT) plays a key role in the metastasis and drug resistance of non-small-cell lung cancer. IGF-1 treatment of A549 cells stimulated the expression of SPHK1.

To conclude, there is promising evidence of in vitro studies that targeting SPHK1 in a chemotherapeutic setting might be of therapeutic relevance. In the present study, we could show an association with prognostic parameters in vivo. Future clinical studies are needed to establish the clinical relevance of sphingolipid metabolism, especially focusing on targeting SPHK1 for chemotherapy-sensitizing effects in ovarian cancer patients.

## Supplementary Information

Below is the link to the electronic supplementary material.

Electronic supplementary material 1 (DOCX 14 kb)

## Data Availability

The datasets used and/or analyzed during the current study available from the corresponding author on reasonable request.
