# Supplementary material for: Sphingosine-kinase-1 expression is associated with improved overall survival in high-grade serous ovarian cancer
Source: J Cancer Res Clin Oncol. 2021 Mar 3;147(5):1421–30. doi: 10.1007/s00432-021-03558-x (PMC8021516; doi:10.1007/s00432-021-03558-x)
Supplement: Supplementary file 1 — Electronic supplementary material 1 (DOCX 14 kb) [file 432_2021_3558_MOESM1_ESM.docx]

**Table S1: Clinical parameters of the analyzed cohorts**

|  | **TMA cohort** |  |  |  |
| --- | --- | --- | --- | --- |
| **Parameter** | **OOU (n=508)** | **OOUE (n=241)** | **VOA (n=256)** | **Overall (n=1,005)** |
| **Age (years)** |  |  |  |  |
| <50 | 150 (29.5%) | 39 (16.2%) | 59 (23.0%) | 248 (24.7%) |
| >50 | 358 (70.5%) | 202 (83.8%) | 197 (77.0%) | 757 (75.3%) |
| **Histology** |  |  |  |  |
| Clear cell OC | 126 (24.8%) | 11 (4.6%) | 28 (10.9%) | 165 (16.4%) |
| Endometroid OC | 127 (25.0%) | 4 (1.7%) | 24 (9.4%) | 155 (15.4%) |
| High-grade serous OC | 203 (40.0%) | 219 (90.9%) | 191 (74.6%) | 613 (61.0%) |
| Mucinous OC | 35 (6.9%) | 4 (1.7%) | 7 (2.7%) | 46 (4.6%) |
| Low-grade serous OC | 11 (2.2%) | 3 (1.2%) | 6 (2.3%) | 20 (2%) |
| Other* | 6 (1.2%) | 0 (0.0%) | 0 (0.0%) | 6 (0.6%) |
| **Debulking surgery** |  |  |  |  |
| Optimal ^$^ | 508(100.0%) | 0 (0.0%) | 90 (35.2%) | 598 (59.5%) |
| Suboptimal ^§^ | 0 (0%) | 241 (100.0%) | 166 (64.8%) | 407 (40.5%) |
| **FIGO stage** |  | n=240 | n=253 | n=1,001 |
| I-II | 424 (83.5%) | 16 (6.7%) | 70 (27.7%) | 510 (51.0%) |
| III-IV | 84 (16.5%) | 224 (93.3% | 183 (72.3%) | 491 (49.0%) |
| **SPHK1 expression** |  |  |  |  |
| low | 386 (76.0%) | 232 (96.3%) | 139 (54.3%) | 757 (75.3%) |
| high | 122 (24.0%) | 9 (3.7%) | 117 (45.7%) | 248 (24.7%) |

^*^ former Transitional cell OC according to WHO classification

^$^ no residual tumor

^§^ any residual tumor
